# Phenotypes in children with *GNAO1* encephalopathy in China

**DOI:** 10.3389/fped.2023.1086970

**Published:** 2023-08-29

**Authors:** Yanmei Li, Hong Chen, Lin Li, Xueyan Cao, Xin Ding, Li Chen, Dezhi Cao

**Affiliations:** ^1^ Shenzhen Children’s Hospital, Shantou University, Shenzhen, China; ^2^ Department of Neurology, Shenzhen Children’s Hospital, Shenzhen, China; ^3^ Surgery Division, Epilepsy Center, Shenzhen Children’s Hospital, Shenzhen, China

**Keywords:** GNAO1, gene, epilepsy, movement disorder, encephalopathy, status dystonicus

## Abstract

**Background:**

The *GNAO1* gene encodes the α-subunit (Gαo) of the heterotrimeric guanine nucleotide-binding protein (G protein). The aim of this study was to explore the clinical characteristics of patients with *GNAO1* pathogenic variations.

**Methods:**

Ten patients with pathogenic variations in *GNAO1* were enrolled from the Shenzhen Children's Hospital. Clinical data from several cases previously reported from China were also included and analyzed.

**Results:**

Twenty-seven patients with variations in *GNAO1* were analyzed (10 patients from Shenzhen Children's Hospital, 17 patients from previously published studies) including 12 boys and 15 girls. The median age of onset was 3 months with moderate to severe global developmental delay. Nineteen different *GNAO1* heterozygous variants were identified. Epilepsy was observed in 18 patients (67%, 18/27), movement disorder (MD) was observed in 22 patients (81%, 22/27), and both were seen in 13 patients (48%, 13/27). Seizures typically presented as focal seizures in all patients with epilepsy. MD typically presented as dystonia and chorea. Loss-of-function (LOF) or partial loss-of-function (PLOF) mutations were more frequent in patients with developmental and epileptic encephalopathy (*p* = 0.029). Interictal electroencephalograms showed multifocal or diffuse epileptiform discharges. The most common magnetic resonance imaging finding was widened extracerebral space. In contrast to MD, in which improvements were not common, seizures were easily controlled by anti-seizure medications. Severe dystonia in three patients was effectively treated by deep brain stimulation. Seven (26%, 7/27) patients died of respiratory complications, status dystonicus, choreoathetosis, or sudden unexpected death in epilepsy.

**Conclusion:**

We analyzed clinical data of 27 cases of *GNAO1*-related encephalopathy in China. MD seemed to be the central feature and was most difficult to control. LOF or PLOF variants were significantly associated with developmental and epileptic encephalopathy. The active intervention of severe dystonia may prevent death due to status dystonicus. However, future studies with larger samples are needed to confirm these results.

## 1. Introduction

The *GNAO1(MIM 139311)* gene maps on chromosome 16q13 and encodes the Gαo subunit of heterotrimeric guanine nucleotide-binding protein (G protein), which is present in many important G protein-coupled receptors, such as γ-aminobutyric acid receptor type B, dopamine receptor D2, and A1 adenosine and α2-adrenergic receptors (1). The typical function of Gαo is the inhibition of cyclic adenosine monophosphate (cAMP). *GNAO1* is widely expressed in the brain, especially in the hippocampus, striatum, and cerebellum, and plays an important role in neural development and synaptic transmission (1, 2). Animal experiments have shown that *GNAO1* knockout mice showed neurological symptoms such as tremors, seizures, abnormal behaviors, and movement disorders (MDs) and often died early (3).

In 2013, *GNAO1-*related encephalopathy was first reported in patients with Ohtahara syndrome and early infantile epileptic encephalopathy 17 (EIEE17; Online Mendelian Inheritance in Man 615473) (2). More recently, about 150 cases of *GNAO1* variants associated with MD with or without seizures have been reported worldwide (4–8). Interestingly, Feng et al. reported a biochemical analysis of 15 different *GNAO1* mutant alleles that revealed that loss-of-function (LOF) *GNAO1* alleles were associated with epilepsy based on an inability to suppress cAMP production, whereas gain-of-function (GOF) or normal-function (NF) *GNAO1* alleles were associated with MDs (9). In addition, developmental and epileptic encephalopathy (DEE) was correlated with LOF variants in a genotype–phenotype association analysis of 58 patients (4).

In the present study, we analyzed the genotypes and clinical phenotypes of ten patients with *GNAO1* gene variants and data from 17 previously reported *GNAO1*-related encephalopathy cases from China (10–15). We analyzed possible genotype–phenotype correlations in patients with *GNAO1* variants based on recent studies on the functional behavior of several *GNAO1* variants (4, 9).

## 2. Materials and methods

### 2.1. Case collection

Ten patients with *GNAO1* encephalopathy were enrolled from September 2020 to November 2022 at Shenzhen Children's Hospital (Shenzhen, China). The study protocol and consent documents were approved by the Institutional Review Board of Shenzhen Children's Hospital (No:202202001). Written informed consent was obtained from the parents or legal guardians of the patients, and assent was obtained when appropriate. Patients were followed-up at the pediatric neurology clinic or via telephone.

### 2.2. Genetic assessment

Blood samples were collected from participants and both of their parents for whole-exome sequencing, whole-genome sequencing, or targeted capture next-generation sequencing. Sanger sequencing was performed for validation. Variant prediction and interpretation followed the American College of Medical Genetics and Genomics guidelines (16). We classified all variants into LOF, partial-loss-of-function (PLOF), GOF, NF, and unknown-function based on the inhibition of cAMP production in Gαo mutants (4, 9). Furthermore, *GNAO*1 variants associated with disease were assessed for pathogenicity using several predictive bioinformatic tools: Mutation Taster (http://www.mutationtaster.org), Polyphen-2 (http://genetics.bwh.harvard.edu/pph2/), and SIFT (http://sift.jcvi.org).

### 2.3. Clinical assessment

Clinical information was collected retrospectively from face-to-face and/or telephone interviews with patients, their families, or treating physicians. A questionnaire was developed to gather detailed information regarding sex, age at onset, family history, pre- and perinatal events, neurological symptoms, psychomotor development, cognitive function, neurological examination, treatments, and outcomes. Magnetic resonance imaging (MRI) and electroencephalography (EEG) data were also collected and reviewed by experienced pediatric neurologists. The follow-up period was terminated on January 30th, 2023 or at death.

### 2.4. Literature review

Previously reported cases in the Chinese population were identified via the China Knowledge Network (https://cnki.net/), Wanfang Data (https://wanfangdata.com.cn/), and PubMed search using the keywords “*GNAO1*” and “China.” Clinical and molecular genetic data were obtained from the respective references after removing duplicate individuals.

### 2.5. Statistical analysis

The clinical data for all patients were collected and analyzed using descriptive statistics. Continuous data are presented as median (range), and categorical data are presented as frequency (percentage). To test whether the associations of symptoms differed between GOF and LOF/PLOF, Fisher's exact test (2 × 2 contingency table) was used to analyze nonrandom associations between two categorical variables. Fisher's exact test was reliable for the small sample size in the present study. Statistical analysis was performed using STATA SE (v16.0; StataCorp, College Station, TX, USA). All statistical tests were two-sided, and *p* < 0.05 was considered statistically significant.

## 3. Results

### 3.1. Demographic information

Ten patients were enrolled including three boys and seven girls. All patients were unrelated with negative family history for developmental delay (DD), epilepsy, or MD. Pregnancy and birth history were unremarkable. In addition, clinical data from 17 patients with *GNAO1* variants from previously published articles were included. A summary of the individuals with *GNAO1* variants is provided in Supplementary Table S1.

### 3.2. Genetic results

**Figure 1:**
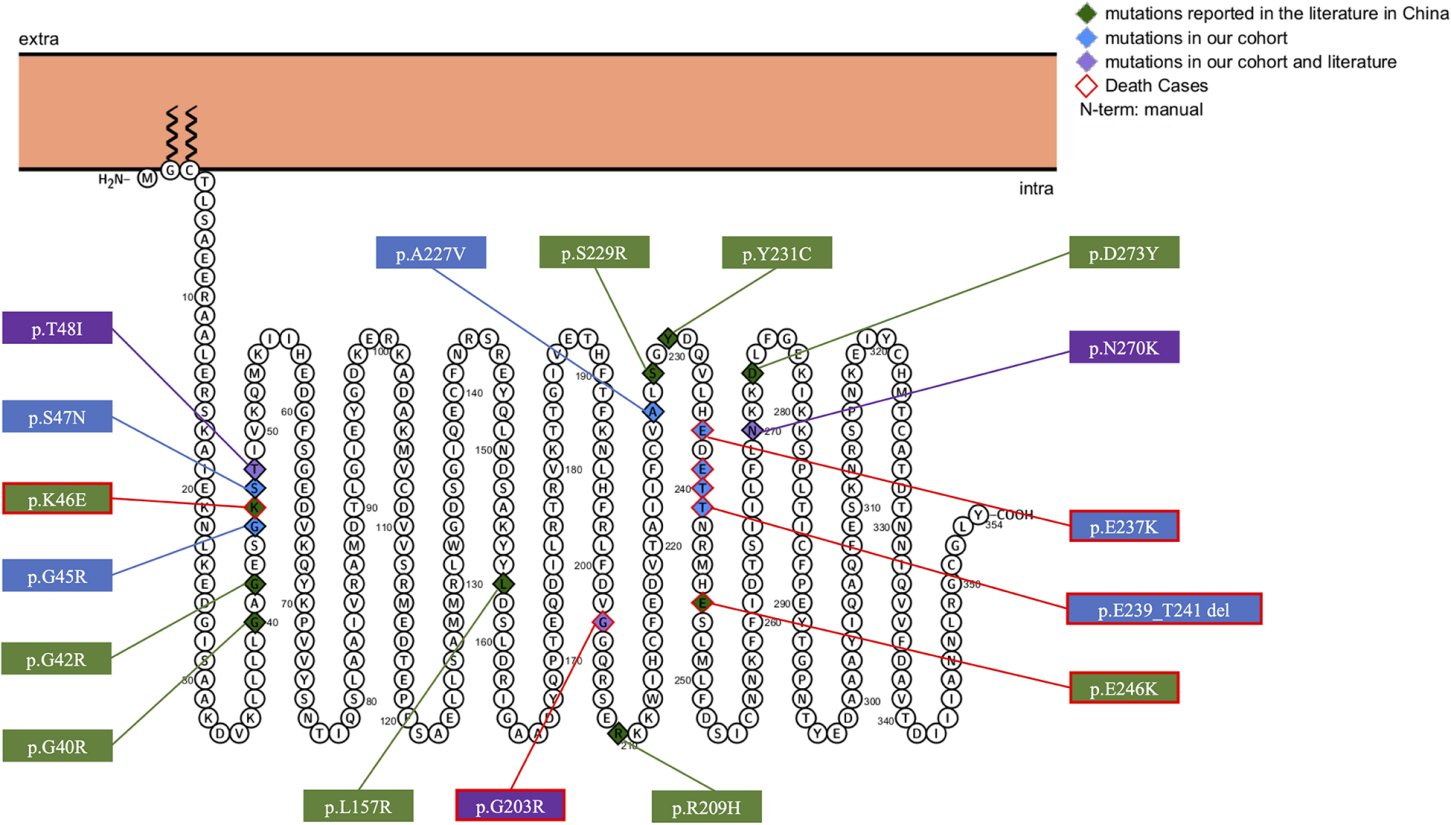
Membrane topology and distribution for *GNAO1* variants predicted by PROTTER online tool (P09471, GNAO_HUMAN).

Eight different variant types were observed in the ten included patients, including six reported variants, c.607G>A(p.G203R), c.709G>A(p.E237K), c.680C>T(p.A227V), c.143C>T(p.T48l), c.808A>G(p.N270D), c.133G>C(p.G45R), and two novel variants, c.140G>A(p.S47N), and c.717_723+1del. We identified 19 different *GNAO1* heterozygous variants, including 17 missense variants, one deletion, and one splicing variant. The variants were found to occur *de novo* after testing the parental DNA in all patients. The most common variants identified were c.607G>A(p.Gly203Arg) (*n* = 4), c.709G>A(p.Glu237Lys) (*n* = 3), c.724-8G>A (*n* = 3), and c.143C>T(p.Thr48Ile) (*n* = 2). Membrane topology and distribution of their *GNAO1* variants were predicted using the PROTTER program (http://wlab.ethz.ch/protter/start/; Figure 1). All variants were tested using the *in silico* variant prediction tools PolyPhen-2, Mutation Taster, and SIFT, and predicted to be pathogenic or likely pathogenic variants. More detailed information on the *GNAO1* gene is shown in Table 1.

**Table 1:** The information of genotype and clinical phenotype in patients associated with *GNAO1* gene mutations in China.

ID	Gender	Onset age		Age at last follow-up	GNAO1 mutations	Mutation type	Pathogenicity	Function of mutation	Seizure types	Epileptic syndrome	MD (symptom)			DD	Interictal EEG	MRI	Treatment	Seizures or MD at last follow-up
		epilepsy	MD								Dystonia	Chorea	Other					
1	F	3m	2y	4y	*c.607G>A* (p.G203R)	Missense	Pathogenic	GOF	FS, GTCS, SE	-	+ (hypotonia)	+	-	delay	MFD, FWR	Widened extracerebral space	VPA, LEV, LD, GBP	Died
2	F	-	8m	2y5m	*c.709G>A* (p.E237K)	Missense	Pathogenic	UF	-	-	+ (appendicular hypertonia)	+	-	delay	N	Widened extracerebral space	None	Died
3	F	31d	5m	4y1m	*c.680C>T* (p.A227V)	Missense	Pathogenic	PLOF	FS, MS, TS	EIDEE	+ (hypotonia, appendicular hypertonia)	-	-	delay	SSW	Widened subdural space	VPA, LEV, PB	Seizure-relief, but dystonia persisted
4	F	4m	1y2m	2y3m	*c.140G>A* (p.S47N)	Missense	Likely pathogenic	UF	FS, GTCS, SE	IESS	+ (axial and appendicular hypertonia)	-	-	delay	MFD, SSW	Subdural effusion, widened extracerebral space	VPA, LEV, TPM, OXC, PB, LTG, PER, VGB, TBZ, KD	FS occurred 2–10 times a day, severe dystonia
5	M	12y	12y	12y9m	*c.717_723+1del*	Deletion	Pathogenic	LOF	FS, TS	-	-	+	orofacial dyskinesia	delay	MFD	Abnormal corpus callosum	VPA, ACTH, CZP, RPD, LD	Died
6	F	30d	6m	2y3m	*c.143C>T* (p.T48I)	Missense	Pathogenic	UF	FS, GTCS	EIDEE	+ (axial and appendicular hypertonia, opisthotonos position)	-	-	delay	MFD, SSW	Arachnoid cyst, Smaller temporal lobe gyrus	VPA, LEV, TPM, PB, CZP, VGB, PER, KD	Seizure-free for 10 months, but MD persisted
7	F	-	5m	4y7m	*c.709G>A* (p.E237K)	Missense	Pathogenic	UF	-	-	+ (thumb adduction)	+	-	delay	N	N	LD	Severe chorea and dystonia
8	M	-	9m	3y7m	*c.709G>A* (p.E237K)	Missense	Pathogenic	UF	-	-	+ (axial and appendicular hypertonia, opisthotonos position)	+	-	delay	N	N	THX, TIZ	Severe chorea and dystonia
9	M	2m	5m	7m	*c.808A>G* (p.N270D)	Missense	Likely pathogenic	LOF	ES, TS, GTCS	IESS	+ (axial and appendicular hypertonia)	-	-	delay	BS, hypsarrhythmia	N	VPA, TPM, LD, VGB, PER, CZP, ATCH	Seizure occurred 2–3 times a day, and dystonia persisted
10	F	5d	2m	1y5m	*c.133G>C* (p.G45R)	Missense	Likely pathogenic	UF	FS, ES, TS	IESS	+ (appendicular hypertonia)	-	-	delay	MFD, SSW, BS	N	VPA, LEV, OXC, VGB, TPM, LTG	Poor seizure control
11 (15)	F	12d	-	1y	*c.607G>A* (p.G203R)	Missense	Pathogenic	GOF	FS, ES	IESS	-	-	-	delay	hypsarrhythmia	N	LEV, VPA, TPM, VGB	Died
12 (14)	M	5m	NA	NA	*c.607G>A* (p.G203R)	Missense	Pathogenic	GOF	FS	-	+	+	Orofacial dyskinesia	delay	MFD, SB	Widened extracerebral space	TPM, VPA, OXC, CZP	Seizure-relief, but MD persisted
13 (12)	F	1m23d	-	5m17d	*c.143C>T* (p.T48I)	Missense	Pathogenic	UF	FS	-	-	-	-	delay	SB, FWR	Increased water content in the white matter	TPM, LEV	Poor seizure control
14 (13)	F	6h	-	1y11m	*c.136A>G* (p.K46E)	Missense	Pathogenic	UF	FS, ES, TS	IESS	-	-	-	delay	FMD, DSW, SB, hypsarrhythmia	N	VPA, TPM, PB, LEV, ACTH	Died
15 (13)	M	1.5m	NA	1y9m	*c.687C>G* (p.S229R)	Missense	Pathogenic	UF	FS, ES, AAS	IESS	+	-	-	delay	hypsarrhythmia, DSW, MFD	White matter delayed myelination	VPA, TPM, ACTH, VGB	Seizure occurred 10 times a day, and dystonia persisted
16 (13)	F	1d	NA	1y5 m	*c.470T>C* (p.L157R)	Missense	Pathogenic	UF	FS, ES	IESS	+	-	-	delay	hypsarrhythmia, BS	N	VGB, TPM, ACTH	Seizure-free, but dystonia persisted
17 (13)	M	4m	-	1y1m	*c.118G>C* (p.G40R)	Missense	Pathogenic	LOF	FS, ES	IESS	-	-	-	delay	hypsarrhythmia, SB, SSW	Widened extracerebral space	TPM, VPA, LEV	Seizure-free
18 (13)	M	9d	NA	1y7m	*c.810C>A* (p.N270K)	Missense	Pathogenic	LOF	FS, ES, TS	IESS	+	-	-	delay	BS, hypsarrhythmia	N	TPM, VPA, VGB, PER, LEV, LCM, PB, ACTH, LD	Seizure occurred 10 times a day, and dystonia persisted
19 (13)	F	2d	NA	3y5m	*c.817G>T* (p.D273Y)	Missense	Pathogenic	UF	FS, GTCS	EIDEE	+	-	-	delay	DSW, SB	N	VPA, LEV, CZP, KD	Seizure-free, but dystonia persisted
10 (13)	F	3d	NA	8m	*c.692A>G* (p.Y231C)	Missense	Pathogenic	PLOF	FS, ES	EIDEE	+	-	-	delay	DSW, FWR, MFD	Widened extracerebral space	VPA, TPM, VGB	Seizure-free, but dystonia persisted
21 (13)	M	12d	NA	10m	*c.607G>A* (p.G203R)	Missense	Pathogenic	GOF	FS	-	+	-	-	delay	MFD	N	TPM, LD	Died
22 (13)	M	-	-	2y3m	*c.736G>A* (p.E246K)	Missense	Pathogenic	GOF	-	-	-	-	-	delay	N	Widened extracerebral space, white matter developmental delay	None	Died
23 (13)	M	-	4m	7y3m	*c.724-8G>A*	Splice site	Pathogenic	UF	-	-	+	-	-	delay	SB	N	LD	MD persisted
24 (10)	F	-	3m	8m	*c.626G>A* (p.R209H)	Missense	Pathogenic	NF	-	-	-	+	-	delay	N	N	None	NA
25 (11)	F	-	6m	6y	*c.124G>A* (p.G42R)	Missense	Pathogenic	GOF	-	-	+	-	trunk torsion	delay	N	N	GPI-DBS	BFMDRS score from 77 dropped to 63 after 24 months, useful grasping
26 (11)	M	-	6m	6y	*c.724-8G>A*	Splice site	Pathogenic	UF	-	-	+	-	trunk torsion	delay	N	N	STN-DBS	BFMDRS score from 62 dropped to 35 after 15 months, walk independently
27 (11)	M	-	2y	18y	*c.724-8G>A*	Splice site	Pathogenic	UF	-	-	+	-	trunk torsion	NA	N	N	LD, CZP, THX, GPI-DBS	BFMDRS score from 86 dropped to 41 after 14 months

m, months; y, years; d, days; h, hours; GOF, gain-of-function; UF, unknown-function; PLOF, partial-loss-of-function; LOF, loss-of-function; NF, normal-function; FS, focal seizure; CTCS, generalized tonic clonic seizure; SE, status epilepticus; MS, myoclonic seizure; TS, tonic seizure; ES, epileptic spasm; AAS, atypical absence seizures; EIDEE, early infantile developmental and epileptic encephalopathy; IESS, infantile epileptic spasm syndrome; MD, movement disorders; DD, developmental delay; MFD, multifocal discharge; FWR, fast wave rhythm; SSW, sharp slow wave; BS, burst-suppression pattern; SB, slow background; DSW, diffuse slow wave; MRI, magnetic resonance imaging; ASMs, anti-seizure medications; VPA, valproate; LEV, levetiracetam; LD, levodopa; GBP, gabapentin; PB, phenobarbital; TPM, topiramate; OXC, oxcarbazepine; LTG, lamotrigine; PER, perampanel; VGB, vigabatrin; TBZ, tetrabenazine; KD, ketogenic diet; ACTH, adrenocorticotropic hormone; CZP, clonazepam; LCM, lacosamide; RPD, risperidone.; THX, trihexyphenidyl; TIZ, tizanidine; GPI-DBS, globus pallidus interna-deep brain stimulation; STN-DBS, subthalamic nucleus-deep brain stimulation; -, absent; NA, not available; N, normal value.

### 3.3. Clinical data

The phenotypes of 27 patients (12 male, 15 female) with *GNAO1* gene variants included 22 (81%, 22/27) patients with MD, 18 (67%, 18/27) patients with epilepsy, and 13 (48%, 13/27) patients with both MD and epilepsy. The median age of onset of symptoms was 3 months (range, 6 h to 4 years). The most common initial symptom was epileptic seizure (63%, 17/27) with a median onset age of 30 days. The median age of symptoms onset for the MD was 6 months. The clinical characteristics of the patients are summarized in Table 1.

MDs presented as mixed hyperkinetic syndrome including dystonia, chorea, and orofacial dyskinesia. The median age of onset of MD was 8 months. Provoking factors for episodes included fever (patients 1 and 5) and emotional stimulus (patients 4, 8 and 9). Dystonia (91%, 20/22) was the most common form of MD, followed by chorea (32%, 7/22). Patients with variant sites in c.709G>A or c.724-8G>A only had MD but not epilepsy. In our cohort, two patients (patients 1 and 3) presented with generalized hypotonia, and patient 3 presented with appendicular hypertonia. Axial and appendicular hypertonia was observed in four patients (patients 4, 6, 8, and 9), with opisthotonos position in patients 6 and 8. Other children with dystonia showed single appendicular hypertonia (patient 2, 10) and thumb adduction (patient 7). Moreover, three patients presented with severe chorea (patients 1, 5, and 8).

Seizures typically presented as focal seizures in all patients with epilepsy, then progressed to multiple seizure types including epileptic spasm (50%, 9/18) and tonic seizures (33%, 6/18). The median age of oneset of epilepsy was 3 months. Two patients had status epilepticus events. Thirteen children developed DEE including nine patients with Infantile Epileptic Spasm Syndrome (IESS), and four with EIDEE. In our cohort, two patients presented with epilepsy in infancy, followed by chorea (patient 1) and dystonia in early childhood (patient 6). For patient 5, delay in motor and cognitive functions during childhood was observed by the parents. However, the patient developed episodic MD after fever at the age of 12, manifested by orofacial dyskinesia with sustained torsional movements of the extremities, and epileptic seizures began 2 months later.

Almost all patients had mild to severe DD, manifesting with motor, speech, and cognitive deficits in the first year of life. In our cohort, seven patients (patient 1, 4, 6, 7, 8, 9 and 10) displayed severe global DD, with absence of head control, absence of speech, and intellectual disability throughout the entire disease course. In the other children, motor and language development, including head control, sitting, standing, walking, and speech skills, were also delayed but were better than in the other seven patients. Moreover, patient 5 was able to stand with help and speak (“ba ba, ma ma”) until the age of 12 years, but was unable to walk independently. However, after the onset of MD, the patient was observed to have development regression manifesting with progressive inability to raise his head stably and loss of language function due to severe dystonia and chorea.

### 3.4. Genotype–phenotype correlations

Genotype–phenotype associations were evaluated. Most of the mutation sites in patients with MD were located within or near the mutational hotspots (207–246 amino acid region). Of the 27 included cases, six carried GOF variants, six PLOF or LOF, one NF, and the remaining 14 carried variants whose functional effects were uncertain. Four clinical features, including epilepsy, DEE, MD, and death as final outcome were used to evaluate associations with different variant functions. LOF or PLOF variants were associated with a higher frequency of DEE compared with GOF or NF variants (*p* = 0.029). However, there were no statistically significant differences between GOF/NF and PLOF/LOF variants regarding epilepsy, MD, and death (Table 2).

**Table 2:** Association between genotype and phenotype in patients carrying different functional variants.

	GOF/NF (*n* = 7)	LOF/POLF (*n* = 6)	*p*
Epilepsy	4	6	0.192
DEE	1	5	**0** **.** **029**
MD	5	5	1.000
Death	4	1	0.266

DEE, development and epileptic encephalopathy; GOF, gain-of-function; LOF, loss-of-function; PLOF, partial-loss-of-function.

The bold font indicates the difference was significant statistically.

### 3.5. Electroencephalography and neuroimaging findings

Interictal EEG showed abnormalities in 19 patients. Focal or multifocal discharges involving the frontal, temporal, or occipital lobe or the central area were seen in all patients with epilepsy. Slow background activity was present in six patients, hypsarrhythmia in seven, and burst-suppression in four. MRI in most individuals showed normal results or no specific findings. Widened extracerebral space was seen in eight patients; other MRI findings included abnormal corpus callosum, arachnoid cysts, smaller temporal lobe gyrus, and white matter abnormalities.

### 3.6. Treatment

The patients were followed-up for at least 3 months, and their ages ranged from 7 months to 18 years at the last follow-up. Among all available medications to control MDs, the most commonly used was levodopa (Table 2); others included gabapentin, tetrabenazine, trihexyphenidyl, and tizanidine. However, these medications did not effectively improve MD symptoms. Three patients (patients 25–27) with MD underwent deep brain stimulation (DBS) (11). Globus pallidus interna (GPI) DBS was performed in patients 25 and 27, and subthalamic nucleus (STN) DBS was performed in patient 26. Following surgery, both GPI and STN DBS were effective in improving dystonia symptoms. Seventeen children were treated with more than two anti-seizure medications (ASMs). The most frequently prescribed ASMs were valproate, topiramate, and levetiracetam. In addition, three patients were prescribed ketogenic diets but discontinued due to poor efficacy. At last follow-up, seizure-free status was obtained by treatment with multiple ASMs in five patients, seizure remission (reduction of seizure frequency >50%) occurred in two patients, and poor seizure control was seen in six patients.

### 3.7. Cause of death

Seven patients (26%) with *GNAO1* variants experienced death (13, 15), 3 patients from Shenzhen Children's Hospital and 4 patients from previously published studies, including 3 boys and 4 girls. Of these deceased patients, the median age at death was 2 years 3 months (ranged 10 months to 12 years 9 months). Three patients had the same variant (p.G203R) located in the hotspot of variants (1). The common causes of death were respiratory complications (3/7) and status dystonicus (SD) (3/7) (Table 3). Another cause of death was sudden unexpected death in epilepsy. Pyrexia/infection is the commonest trigger for SD. In our cohort, Patient 1 and 2 presented with exacerbations of dystonia that were triggered by infection, lasting for hours to days ending up in dystonic state with rising creatine kinase and renal failure causing multiple organ dysfunction and death. Due to the young age, we were not able to perform deep brain stimulation (DBS). Patient 5 experienced hyperkinesia with dystonia and continuous choreoathetosis of the limbs and trunk at 12 years old. At age 12 years 9 months, he developed dyskinetic state triggered by an infection, requiring intensive care, with deep sedation. SD persisted, with rise in creatine kinase, leading to multisystemic deterioration and death.

**Table 3:** Causes of death in patients with *GNAO1* gene variants.

ID	Gender	Onset age	Age at death	Provoking factor	Immediate cause of death	Underlying cause of death
1	F	3m	4y	Pyrexia	Renal failure due to rhabdomyolysis	Status dystonicus
2	F	8m	2y5m	Pyrexia	Renal failure due to rhabdomyolysis	Status dystonicus
5	M	3m	12y9m	Pyrexia	Renal failure due to rhabdomyolysis	Status dyskinetic
11 (15)	F	12d	1y	Pyrexia and cough	NA	Severe pneumonia
14 (13)	F	6h	1y11m	NA	NA	SUDEP
21 (13)	M	12d	10m	NA	NA	Milk choking and asphyxia
22 (13)	M	4m	2y3m	NA	NA	Severe pneumonia and respiratory failure

F, female; M, male; m, months; d, days; h, hours; y, years; NA, not available; SUDEP, sudden unexpected death in epilepsy.

## 4. Discussion

Gαo, encoded by the *GNAO1* gene, could be involved in signal transduction processes of G proteins. Gα binds with Gβγ and GDP in its initial state. Upon activation, Gα exchanges GDP for GTP. Both the active Gα–GTP and Gβγ subunits carry out separate downstream signaling (17). Many downstream targets of Gαo could lead to symptoms of epilepsy or MDs, including inhibiting the expression of adenylyl cyclase, which decreases cyclic cAMP production; N-type (Cav2.2) and P/Q type calcium channel (Cav2.1) activation; and stimulating the opening of inward rectifying potassium channels (1). cAMP signaling and neurotransmitter release affect many ongoing neural functions and neurological development (18). This could explain why most patients with *GNAO1* variants exhibit DD.

Data were available on 27 patients with *GNAO1* variants from China. The age of onset in most individuals was infancy. Our study showed that the DD phenotype was seen in almost all patients, consistent with previous reports (4, 8). MD seemed to be the central feature of *GNAO1* encephalopathy with high incidence (81%), and it was more difficult to control (4–6). The symptoms of attacks included dystonia, chorea, and orofacial dyskinesia. Triggers of attacks included fever and emotional agitation. In a recent article including 157 cases with *GNAO1* variants, *GNAO1*-related MD usually starts during infancy or childhood, with a median age at onset of 26 months (8). We observed that the age of onset of MD was 8 months, which is earlier than previously published cases. At last follow up we found no parkinsonian features and ocular movement abnormalities which Novelli and colleagues reported (8). More than half of patients (67%) experienced seizures with multiple seizure types including focal seizures, tonic seizures, epileptic spasm, and myoclonic seizures. Among them, focal seizures were the most common seizure type. Epilepsy in the present cases was more easily controlled compared to MD, consistent with previous reports (19). *GNAO1* encephalopathy was associated with IESS and EIDEE. EEGs were characterized by focal and multifocal epileptiform discharges, generalized sharp slow waves, hypsarrhythmia, and slow activity. However, the pathogenic variant frequency of *GNAO1* in the Chinese population patients is still unknown due to a lack of large multicenter prospective cohort studies.

In our genotype–phenotype correlation study, we found that LOF or PLOF variants were associated with DEE, which is in agreement with previous observations (4). However, we found no statistical correlation between GOF variants and MD symptoms. Considering the small sample size collected herein, further study with a larger sample of patients with *GNAO1* encephalopathy will be required to confirm the observed genotype–phenotype correlations. In a previous study, LOF variants, mostly involving GTPase, led to enhanced cAMP-mediated signaling and calcium channel activity, increasing excitatory neurotransmission and neuron hyperexcitability, which may explain the pathogenesis of *GNAO1*-related epilepsy. On the contrary, GOF variants, near the ribose and phosphate moieties of the bound GDP, may reduce the release of neurotransmitters, leading to MD (1, 9). However, gene function is still unknown in about half of the patients. Obviously, regarding the biological activity of the *GNAO1* gene, the ability to suppress cAMP production may not be present in all cases. Regarding striatal pathways, another mechanism to explain MDs relies on modifying inhibitory and stimulatory G protein-coupled receptor signaling to cAMP in Gαo. In addition, a model has been proposed through a combination of two principal mechanisms in *GNAO*1 disease, loss of signaling ability and dominant-negative interference (20).

Brain MRI showed no structural alterations and displayed widened extracerebral space in many patients with *GNAO1* encephalopathy. According to most findings reported in the literature, brain MRI in many patients demonstrated delayed myelination, corpus callosum dysplasia, and brain atrophy (6, 8, 21). Symptoms worsen with age in some patients, accompanied by progressive brain atrophy (6, 21, 22). An autopsy was performed on a deceased child with the findings of periventricular gliosis, suggesting that a chronic neurodegenerative process could happen in patients with worsening MD (21).

In the data analyzed in the present study, nineteen patients with epilepsy received treatment with a combination of multiple ASMs. The most frequent ASMs were valproate, levetiracetam, and topiramate. For most of the patients, seizure remission was obtained with varying degrees of efficacy. Because norepinephrine-induced calcium-current inhibition is mediated by Gαo, Nakamura et al. suggested that epileptic seizures associated with *GNAO1* variants might be improved by calcium-channel modulators such as pregabalin and gabapentin, which act as selective calcium-channel blockers, and topiramate, which modulates high-voltage-activated calcium channels in dentate granule cells (2). On the contrary, almost all patients with MD did not respond well to treatment. It has been reported that tetrabenazine and DBS were effective therapies for the treatment of MD associated with *GNAO1* (13, 21–28). Tetrabenazine can deplete multiple amine neurotransmitters (dopamine, norepinephrine, and serotonin). Moreover, variable effects on MDs have been reported with gabapentin, topiramate, and levodopa (19, 21, 22, 29). Based on the fact that Gαo has been implicated in the regulation of many signals involved in epilepsy and MDs, it has been proposed that different approaches to therapy for different variants (agonists for LOF and antagonists for GOF mutants) may be a new therapeutic target for patients with *GNAO1* variants (9). Also, while several drugs have shown efficacy in controlling symptoms of epilepsy or MDs, none of these drugs appeared to be able to alleviate developmental delays.

Among the cases of *GNAO1* encephalopathy reported in China, three patients were treated with DBS (11). Patient 26 was first reported to receive STN DBS, and the postoperative Burk–Fahn–Marsden Dystonia Rating Scale score improved by 32% compared with the preoperative score, suggesting that STN may be used as the target of DBS (11). It has been previously reported that it is difficult to control status dystonicus even after one or more doses of drugs or sedatives (19, 22, 25). The fundamental goal of DBS is to alter pathological neural activity within the basal ganglia–thalamocortical circuit. About 20 patients reported a reduced frequency and severity of MD exacerbations after GPI DBS (19, 22–28). Patients undergoing GPI DBS were followed-up for 8–10 years by Koy et al. (22) and Benato et al. (24). The results showed that MD symptoms were controlled well and there were no more episodes of status dystonicus, which demonstrated that DBS had a potential role to control and prevent status dystonicus.

Patients with *GNAO1* pathogenic variants showed poor prognosis with a high mortality rate (26%). Seven patients died of SD and respiratory complications in individuals with *GNAO1* variants in China. To date, 14 patients with variants died because of MD exacerbations, respiratory failure, and neurological deterioration (8). Several cases with similar causes of death have also been reported in the literature (2, 19, 21). Hence, we believe that SD and respiratory complications may be the most common causes of death. Pyrexia/infection is the most provoking factor. Therefore, it is important to prevent infections in children with drug-refractory MD. The active intervention of severe dystonia may prevent death due to SD. DBS has been shown to control and prevent refractory hyperkinetic crises, which can improve prognosis by reducing the length of hospitalization and preventing complications associated with disease and treatment (22, 24, 26). However, generally accepted inclusion criteria for DBS included unequivocal diagnosis of dystonia, medical treatment failure, and sufficient disability to the patient with more than 7 years old (30). For young children (less than 7 years old), it is worth thinking about considering early DBS for patients with drug-refractory MD. Finally, particular attention should also be paid to motor and speech development, mental health, and nutritional status.

## 5. Conclusions

In the present study, our data support the findings that patients with *GNAO1* variants usually present with MD, epilepsy, and DD at varying degrees. The age of onset in most individuals was infancy. MD seemed to be the central feature of *GNAO1* encephalopathy, with an earlier age of onset than previously reported. LOF or PLOF variants were significantly associated with DEE. SD and respiratory complications were the main cause of *GNAO1*-related death. Further studies with a larger number of patients are needed to evaluate the possible genotype–phenotype correlations.

## Data Availability

The datasets presented in this study can be found in online repositories. The names of the repository/repositories and accession number(s) can be found in the article/**Supplementary Material**.
