# Supplementary material for: Phenotypes in children with GNAO1 encephalopathy in China
Source: Front Pediatr. 2023 Aug 29;11:1086970. doi: 10.3389/fped.2023.1086970 (PMC10495587; doi:10.3389/fped.2023.1086970)
Supplement: Supplementary file 1 [file Table1.docx]

| **Gender composition** | |
| --- | --- |
| Male | 12(44%, 12/27) |
| Female | 15(56%, 15/27) |
| **Function of mutations** | |
| GOF | 6(22%, 6/27) |
| LOF/PLOF | 6(22%, 6/27) |
| NF | 1(4%, 1/27) |
| UF | 14(52%, 14/27) |
| **Types of seizure** | |
| Focal seizure | 17(94%, 17/18) |
| Generalized tonic clonic seizure | 5(28%, 5/18) |
| Myoclonic seizure | 1(6%, 1/18) |
| Tonic seizure | 6(33%, 6/18) |
| Epileptic spasm | 9(50%, 9/18) |
| **Epilepsy syndrome** | |
| EIDEE | 4(22%, 4/18) |
| IESS | 9(50%, 9/18) |
| **Types of movement disorders** | |
| Dystonia | 20 (91%, 18/22) |
| Chorea | 6(27%, 6/22) |
| Orofacial dyskinesia | 2(9%, 2/22) |
| Trunk torsion | 3(14%, 3/22) |
| **The surgical treatment** | |
| GPI-DBS | 2(7%, 2/27) |
| STN-DBS | 1(4%, 1/27) |
| **Follow-up data at last time** | |
| Seizure-free | 5(28%, 5/18) |
| Partial seizure control | 2(11%, 2/18) |
| Poor seizure control | 6(33%, 6/18) |
| Efficient control of movement disorders | 2(9%, 2/22) |
| Poor control of movement disorders | 15(68%, 15/22) |
| Died | 7(26%, 7/27) |

**Supplementary Materials**

Supplementary Table 1. Summary of included individuals with GNAO1 variants.
